# A scoping review of programme specific mammographic breast density related guidelines and practices within breast screening programmes

**DOI:** 10.1016/j.ejro.2023.100510

**Published:** 2023-08-02

**Authors:** Jessica O’Driscoll, Aileen Burke, Therese Mooney, Niall Phelan, Paola Baldelli, Alan Smith, Suzanne Lynch, Patricia Fitzpatrick, Kathleen Bennett, Fidelma Flanagan, Maeve Mullooly

**Affiliations:** a School of Population Health, RCSI University of Medicine and Health Sciences, Beaux Lane House, Mercer St. Lower, Dublin 2, Ireland; b National Screening Service, Kings Inn House, 200 Parnell Street, Dublin 1, Ireland; c BreastCheck, National Screening Service, 36 Eccles Street, Dublin 7, Ireland; d School of Public Health, Physiotherapy and Sports Science, University College Dublin, Belfield, Dublin 4, Ireland

**Keywords:** Mammographic breast density, Breast cancer screening programme, Evidence-based guidelines, Scoping review

## Abstract

**Introduction:**

High mammographic breast density (MBD) is an independent breast cancer risk factor. In organised breast screening settings, discussions are ongoing regarding the optimal clinical role of MBD to help guide screening decisions. The aim of this scoping review was to provide an overview of current practices incorporating MBD within population-based breast screening programmes and from professional organisations internationally.

**Methods:**

This scoping review was conducted in accordance with the framework proposed by the Joanna Briggs Institute. The electronic databases, MEDLINE (PubMed), EMBASE, CINAHL Plus, Scopus, and Web of Science were systematically searched. Grey literature sources, websites of international breast screening programmes, and relevant government organisations were searched to identify further relevant literature. Data from identified materials were extracted and presented as a narrative summary.

**Results:**

The search identified 78 relevant documents. Documents were identified for breast screening programmes in 18 countries relating to screening intervals for women with dense breasts, MBD measurement, reporting, notification, and guiding supplemental screening. Documents were identified from 18 international professional organisations with the majority of material relating to supplemental screening guidance for women with dense breasts. Key factors collated during the data extraction process as relevant considerations for MBD practices included the evidence base needed to inform decision-making processes and resources (healthcare system costs, radiology equipment, and workforce planning).

**Conclusions:**

This scoping review summarises current practices and guidelines incorporating MBD in international population-based breast screening settings and highlights the absence of consensus between organised breast screening programmes incorporating MBD in current breast screening protocols.

## Highlights

• Differing practices relating to MBD in breast screening settings were found.
• Different supplemental screening methods were identified for women with dense breasts.
• Ongoing studies will further establish the optimal role of MBD in breast screening.

## 1 Introduction

GLOBOCAN estimates show that breast cancer was the most commonly diagnosed cancer type and a leading cause of cancer deaths globally among females in 2020 [1]. Breast cancer incidence is expected to increase worldwide by approximately 41% by 2040 [2]. Breast cancer screening aims to reduce mortality from breast cancer and a recent systematic review by Canelo-Aybar et al. [3], for the European Commission Initiative on Breast Cancer (ECIBC) that included six randomised clinical trials, found that organised breast screening reduced breast cancer mortality for women aged 50–69 years at average risk of breast cancer (Relative Risk (RR): 0.77; 95% CI: 0.66–0.90) invited to attend mammography screening compared to women not invited. As of 2017, 25 European Union (EU) member states have population-based breast screening programmes (BSPs) [4]. Outside the EU, in the United States of America (USA), for example, there is no centrally organised approach and breast cancer screening tends to occur on an individual basis through a range of clinical settings in the different states [5].

One established independent risk factor for breast cancer is mammographic breast density (MBD) which collectively represents the amount of fibroglandular (dense) tissue in the breast [6]. It appears radiologically as white areas on a mammogram. Meta-analysis findings show that women with the highest levels of MBD have a four to six fold increased risk of breast cancer compared to women with the lowest levels of MBD [7]. In addition, higher levels of MBD can lower the sensitivity of mammography to detect breast cancer [6]. Due to these associations, there have been increased discussions surrounding the use of MBD to help guide screening decisions, including for risk stratification, in the breast screening setting [8], [9].

In the USA, state-specific legislation introduced in 38 states requires women to be informed and/or notified about their MBD [10]. Federal legislation updated in 2023 by the Food and Drug Administration now requires the assessment of MBD for women who attend for breast screening in the USA and for these women to be notified regarding their breast density from September 2024 [11]. Outside of the USA, discussions regarding the notification of women who attend routine breast screening about their MBD have led to BSP specific guidelines being introduced in parts of Australia and Canada [8], [12], [13]. Recently, the European Society of Breast Imaging (EUSOBI) published recommendations for women with extremely dense breasts, classified according to the American College of Radiology (ACR) Breast Imaging-Reporting and Data System (BI-RADS) atlas, which included informing women about their MBD, the implications of dense breasts and if supplemental magnetic resonance imaging (MRI) screening or ultrasound in combination with mammography (if MRI is unavailable) was indicated [14]. Furthermore, the guideline development group (GDG) of the ECIBC stated that ‘information and education for women about MBD is critical’ [15]. Currently, there is no guideline from the ECIBC recommending that organised BSPs should notify women about MBD.

In relation to MBD and supplemental investigations, imaging modalities such as MRI and ultrasound have shown higher cancer detection rates. These modalities however also have limitations, such as higher rates of false positive results than mammography [16], [17], [18]. Recent published evidence syntheses by the ECIBC ”suggest not implementing supplemental screening with MRI, automated breast ultrasound system or hand-held ultrasound (where it is not already in practice) for asymptomatic women with high MBD attending organised screening programmes” [19], [20], [21], [22]. Results, including long term findings, from ongoing clinical trials such as the Dense Tissue and Early Breast Neoplasm Screening (DENSE) trial in the Netherlands, which showed increased cancer detection following supplemental MRI among women with extremely dense breasts, are important to help understand the additional clinical value of using MBD assessment to guide breast screening decision making including the use of supplemental screening for women with dense breasts in an organised screening setting [23], [24], [25].

As the discussion on the incorporation of MBD into population-based breast screening continues, the aim of this scoping review was to describe current guidelines and practices incorporating MBD within population-based BSPs and from professional organisations internationally.

## 2 Material and methods

This scoping review was performed according to a protocol developed a priori informed by the Joanna Briggs Institute (JBI) methodological guidance for scoping reviews [26] outlined in Online Resource 1. This scoping review is reported in accordance with the Preferred Reporting Items for Systematic Reviews and Meta-Analyses extension for Scoping Reviews (PRISMA-ScR) guidelines, the checklist of which is shown in Online Resource 2 [27].

### 2.1 Research question

Guided by the Population, Concept and Context mnemonic [26], the primary research question identified was:

“What are the current programme specific MBD-related guidelines and practices (Concept) for women attending routine breast screening (Population), within organised BSPs (Context)?”.

### 2.2 Search strategy

A comprehensive search strategy was performed as recommended in the JBI guidance [26] and outlined in Online Resource 1. The final search strategy was developed and refined in collaboration with a medical librarian and took place from October to December 2021. The following electronic databases were searched without date restrictions: MEDLINE (PubMed), EMBASE, CINAHL Plus, Scopus, and Web of Science. The search strategies for the databases are provided in Online Resource 3. It was anticipated that information specific to international BSPs may not be fully captured through the electronic database search, therefore, a grey literature search was also conducted from December 2021 to March 2022. The following grey literature databases were searched: OpenGrey.eu, National Institute for Health and Care Excellence (NICE) evidence, and Lenus, the Irish Health Repository. Search engines including Bielefeld Academic Search Engine, Mednar, and the custom Google search engine for official government resources provided by the University of Toronto Libraries were searched. A targeted search of the websites of selected international BSPs and government organisations was also performed to capture information from BSPs not found through the previous searches. Efforts were made to adequately represent countries and regions with organised BSPs within and outside Europe. Reference lists of included documents were screened to identify any further relevant articles.

### 2.3 Eligibility criteria

To address the aim of this review, screening practices, guidelines, or position statements developed by organised BSPs or government agencies were considered when identified. In addition, guidance developed by professional societies were also included. Documents were required to include information relating to screening guidance for the purpose of MBD assessment, reporting, notifying, or for guiding supplemental screening to be selected. Documents were included if they were published up until the time of the electronic database and grey literature searches. Where revisions of documents were found, the most recent version available at the time of the search was selected. The applied inclusion and exclusion criteria is listed in Online Resource 1.

### 2.4 Selection of studies

The identified literature were imported into the online systematic review management tool, Rayyan, [28] and duplicates were removed. Title and abstracts were screened independently by two reviewers using the inclusion criteria (Online Resource 1). Selected literature underwent full text review by one reviewer, and a second reviewer independently screened a random 20% of the identified literature. Any disagreements were resolved by consensus and if necessary, a third reviewer was consulted.

### 2.5 Data extraction

Data was extracted from included documents using a pre-piloted data extraction form developed by the JBI [26] and adapted for this review. Data was collected from the identified literature according to the following headings:

1. Study details: Title, author and year of publication.
2. BSP characteristics: Type of BSP setting (population-based, regional, or organised), location, date of programme commencement, screening interval, screening method, use of double reading, affordability (free, covered by public health insurance, or co-payment), individual invitation, age standardised incidence rate, attendance rate, screening coverage, and recall rate.
3. Population: Target age range and risk profile (average or intermediate risk).
4. Concept: Guidance type (BSP, government agency, or professional society), guidance development methods/source, screening guidance for: dense breasts, MBD assessment, MBD reporting, MBD notification, supplemental screening, and development/implementation considerations for such guidance.

### 2.6 Data collation and reporting

The descriptive summary aligned the summarised results with the scoping review objective and research questions. MBD-related guidelines and practices for BSPs and recommendations/positions from professional societies were sub-divided into those relating to MBD assessment, reporting, notification, or for guiding supplemental screening and the country of each BSP was noted.

## 3 Results

### 3.1 Description of search results

**Fig. 1:**
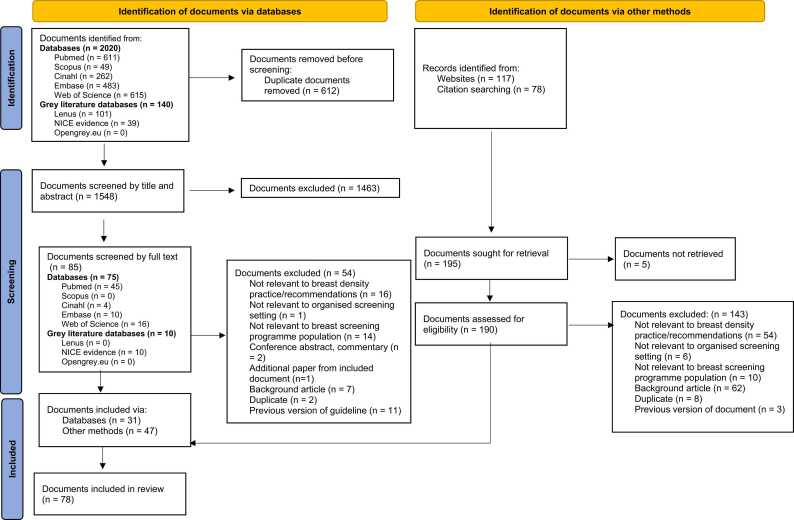
PRISMA flowchart of the document selection process.

A summary of the records identified are shown in Fig. 1. A total of 2160 records were identified from the database searches, with 1548 records remaining after 612 duplicate records were removed. Upon completion of title and abstract screening, 85 documents remained for full text review, after which, 31 documents from the database searches were deemed eligible for inclusion. 195 documents were identified from the targeted search of websites of selected international BSPs and government organisations and from citation searching. Upon full review, 47 documents were included. In total, 78 relevant documents were included in this scoping review.

### 3.2 Characteristics of BSPs for countries where relevant MBD practice related documentation was identified

Relevant documents for the research question from BSPs were identified from 18 countries, with 13 countries located in Europe and 5 countries located outside of Europe (Table 1). 11 of the BSPs were population based, with 7 of these BSPs located in Europe. The target age was programme dependent and ranged between 40 and 75 years, with the BSP in Austria offering an option to voluntarily enrol after screening participants reached the upper end of the target age range [29]. In addition, in BSPs in Austria and Australia, participants below the lower end of the target age range, typically ranging between 40 and 49 years, can also voluntarily partake [29], [30]. In the majority of BSPs, the screening interval was two years between screening phases with the exception of Canada, Sweden, and the UK, which have screening intervals of one or two years (region dependent), 18 months or two years (age dependent), and three years respectively [31], [32], [33]. In all BSPs where documents were identified, digital mammography was the primary screening modality used. In the Czech Republic, ultrasound was also noted as a screening modality used in combination with digital mammography [34].

**Table 1:** Characteristics of BSPs for countries where relevant MBD practice related documentation were identified in the database and targeted searches.

	Country	Type of screening programme	Programme start year	Target age (years)	Screening interval (years)	Screening test
Within the continent of Europe	Austria [29], [47], [48], [49]	PB	2014	45–69 (40–44 and >70 can voluntarily enrol)	2	DM
	Czech Republic [34]	PB	2002	≥45	2	DM, US
	Denmark[35]	OR RB	1991 (Copenhagen), 1993 (County of Funen), 2007 (Nationwide)	50–69	2	DM
	Finland [93]	RB	1987	50–69	2	DM
	France [65]	OR RB	1994	50–74	2	DM
	Germany [74]	PB	2005	50–69	2	DM
	Greece	-	-	-	-	-
	Ireland [94]	PB	2000 (Full national expansion in 2007)	50–69	2	DM
	Italy [95]	RB	-	50–69	2	DM
	The Netherlands [68]	PB	-	50–75	2	DM
	Spain [96]	RB	1990 (Phased), 2006 (Nationwide)	50–69	2	DM
	Sweden [32]	PB	1974 (Phased), 1997 (Fully implemented)	40–74	1.5 (40–54 years), 2 (55–74 years)	DM
	The UK [33]	PB	-	50–70	3	DM
Outside the continent of Europe	Australia [30]	PB	1991 (Phased), 1995 (Fully implemented)	50–74 (40–49 can also register)	2	DM
	Canada [41]	PB	-	40–74 (Region dependent)	1 or 2 (Region dependent)	DM
	Korea [97]	-	1999	40–69	2	DM
	New Zealand [71]	PB	1998	45–69	2	DM
	Taiwan [51]	PB	-	40–70	2	DM

**Where information was not identified a dash (-) was used**, the following abbreviations were also used: PB = Population-based, RB = Regional-based, OR = Organised, DM = Digital Mammography, US = Ultrasound

### 3.3 Summary of MBD-related practices from BSPs

Information relating to MBD practices from BSPs is shown in Table 2. The identified MBD literature were published between 2012 and 2022. Region specific information was found within Australia, Canada, Denmark, and Spain [12], [35], [36], [37], [38], [39], [40], [41], [42], [43], [44], [45]. Literature that mentioned a screening interval specifically for women with dense breasts was found in Sweden and Canada [41], [44], [45], [46]. Literature that mentioned MBD assessment was found in 11 countries, MBD reporting in 7 countries, MBD notification in 4 countries, and for guiding supplemental screening in 14 countries. Four of the countries that assessed MBD referred to the use of the ACR BI-RADS Breast Composition Categories (Version 4 or 5). Ultrasound (n = 11) was the screening modality mentioned most frequently in information relating to supplemental screening for women with dense breasts (Table 2).

**Table 2:** Summary of region and/or country specific information relating to international MBD related screening practices for BSPs.

	Region (If applicable), Country	MBD specific screening interval - Yes/No/Other, (Year of publication)	MBD assessment - Yes/No/Other, (Year of publication)	MBD reporting - Yes/No/Other, (Year of publication)	MBD notification - Yes/No/Other, (Year of publication)	Guiding supplemental screening - Yes/No/Other, (Year of publication)	Additional information relating to MBD assessment, reporting and notification	Supporting material from which material was sourced and collated
Within the continent of Europe	Austria	-	Yes* (2017)	Yes^#^ (2017)	-	Yes^+^ (2022)	*Use ACR classification, ^#^MBD categorisation must be included, ^+^Supplemental US possible for MBD categories C and D	Screening programme website [29], Peer-reviewed publications [48], [49], Government agency quality standard [47]
	Czech Republic	-	Yes (2021)	Yes (2021)	-	Yes* (2021)	*Supplementary US/DBT for women with dense breasts	Screening programme website [34], Ministry of Health document [50]
	Capital Region, Denmark	-	Yes (2012/2013)	-	-	-	Since November 2012, ACR BI-RADS version 4 used to categorise MBD.	Peer-reviewed publication [35]
	Finland	-	-	-	-	No (2021)	Further research needed as current evidence does not allow recommendation of SS methods	National cancer registry report [93]
	France	No (2014)	-	-	-	Yes* (2019)	*Supplemental US performed by radiologist if there is difficulty interpretating mammogram due to the masking effect of MBD	French national authority recommendation [65], Peer-reviewed publication [62]
	Germany	-	No (2017)	-	No (2017)	Other* (2018)	*Need to cover cost of additional MRI or US examinations as not part of screening programme, benefits of these examinations not proven	National HTA body document [63], Screening programme website [67]
	Greece	-	-	-	-	Yes (2019)	Physician performed US for women with extremely dense breasts	Peer reviewed publication [62]
	Ireland	-	No (2021)	-	No (2021)	-	No evidence-based guidance for PB screening programmes women with dense breasts, continuing to follow European guidance	National screening programme document [94]
	Italy	-	-	-	-	No (2022)	Conditional recommendation against the use of supplemental automated breast US, manual US, hand-held US, and MRI.	National screening observatory document [95]
	The Netherlands		No (2022)	No (2022)	-	No* (2022)	*Recommend CEM at the earliest opportunity.	Independent government and parliament scientific advisory body publication [68], Independent agency of the Ministry of Health, Welfare and Sport website [64], [98]
	Madrid, Spain	-	-	-	-	Yes (2017)	Supplementary US/DM if women have dense breasts	Regional government website [36]
	Sweden	Yes (2014)	-	-	-	-	Every 18 months for women in younger age range as they are more likely to have dense breasts	Government agency recommendation [46]
	UK	-	No* (2019)	No (2019)	-	No^#^ (2019)	*No gold standard measurement method, ^#^Insufficient evidence that US reduces interval cancers, mortality, that it is cost effective, and what proportion of additional detected cancers represent over diagnosis.	National screening committee document [54]
Outside the continent of Europe	Australia	-	No (2020)	No (2020)	No (2016)	No (2020)	-	Screening position statement [99], Peer reviewed publication [37]
	Western Australia, Australia	-	Yes* (2020)	Yes^#^ (2020)	Yes^+^ (2020)	No (2020)	*Dichotomous measurement, women with BI-RADS category 3 or 4 = dense, ^#^Flagged by at least one radiologist, ^+^Women are notified if MBD >50 %.	Screening programme document [38], Pre-print publication [39], Peer reviewed publications [12], [37], [40]
	Alberta, Canada	-	Yes (2021)	-	Yes (2021)	Other* (2021)	*May be offered supplemental US if women have dense breasts	Screening programme document [42], Peer reviewed publication [41]
	British Columbia, Canada	-	Yes* (2021)	-	Yes^#^ (2021)	Other - Insufficient evidence^+^ (2021)	*MBD categorised using ACR BI-RADS categories, ^#^Since mid-October 2018, all screening mammogram results include BI-RADS MBD assessments, ^+^Do not recommend supplemental US for women with dense breasts but have access to government funded US if want to access them after a consultation with healthcare provider	Screening programme document [43], Peer reviewed publications [12], [41]
	Manitoba, Canada	No (2021)	-	-	Yes (2021)	-	^-^	Peer reviewed publication [41]
	New Brunswick, Canada	No (2021)	-	-	Yes (2021)	-	^-^	Peer reviewed publication [41]
	Newfoundland and Labrador, Canada	Yes* (2021)	-	-	Yes^#^ (2021)	-	*Annual screening for women with dense breasts, ^#^Women notified if MBD ≥75 % (BI-RADS D)	Peer reviewed publication [41]
	Northwest Territories, Canada	No (2021)	-	-	Yes (2021)	-	If MBD ≥75 % (BI-RADS D)	Peer reviewed publication [41]
	Nova Scotia, Canada	No (2021)	-	-	Yes (2021)	-	^-^	Peer reviewed publication [41]
	Ontario, Canada	Yes* (2021)	-	-	Yes^#^ (2021)	-	*Annual screening for women with dense breasts, ^#^Women notified if MBD ≥75 % (BI-RADS D)	Peer reviewed publications [41], [44], [45]
	Prince Edward Island, Canada	Yes* (2021)	-	-	Yes (2021)	-	*Annual screening for women with dense breasts	Peer reviewed publication[41]
	Québec, Canada	No (2021)	Yes* (2016)	Yes^#^ (2016)	No (2021)	-	*MBD classified using ACR BI-RADS categories, ^#^Provided for every mammogram	Peer reviewed publications [41], [45]
	Saskatchewan, Canada	Yes* (2021)	-	-	Yes^#^ (2021)	-	*Annual screening for women with dense breasts, ^#^Women notified if MBD ≥75 % (BI-RADS D)	Peer reviewed publication [41]
	Yukon Territory, Canada	No (2021)	-	-	No (2021)	-	^-^	Peer reviewed publication [41]
	Korea	-	-	-	-	Other - Insufficient evidence (2015)	Do not recommend or oppose use of US for asymptomatic women	Peer reviewed publication [97], Government institution recommendation [100]
	New Zealand	-	No (2019)	-	-	No (2019)	Insufficient evidence to recommend supplemental US/MRI screening for women with dense breasts and otherwise an average breast cancer risk	Breast screening programme document [71], National screening unit position statement [72]
	Taiwan	-	Yes (2019)	Yes (2019)	-	-	Mammography gland composition included in mammography report with categories – Fatty breast, scattered fibroglandular density, heterogeneously dense, or extremely dense	Government agency document [51]

**Where information was not identified a dash (-) was used**. The following abbreviations were also used: ACR = American College of Radiology, MBD = Mammographic breast density, SS = Supplemental screening, PB = Population-based, HCPs= Healthcare professionals, CEM= Contrast enhanced mammography, DM = Digital Mammography, US = Ultrasound, DBT = Digital Breast Tomosynthesis

### 3.4 Summary of MBD-specific guidance from professional societies and GDGs

A total of 27 relevant documents were identified from 18 professional societies and GDGs as shown in Table 3. The literature identified was grouped according to tailored screening for MBD (n = 2), a screening interval specifically for women with dense breasts (n = 1), MBD assessment (n = 1), MBD reporting (n = 1), MBD notification (n = 3), and guiding supplemental screening (n = 20). Within the guiding supplemental screening category, ultrasound (n = 14) and MRI (n = 8) were the screening imaging modalities mentioned most frequently in the identified documents.

**Table 3:** Summary of MBD related screening recommendations/guidelines/position statements from professional organisations and guideline development groups.

	Professional Organisation & Type of recommendation (if provided)	Year	Recommendation and specific comments
Within the continent of Europe	European Commission Initiative on Breast Cancer Guideline Development Group GDG [15], [19], [20], [21], [22]	2020	Tailored screening for MBD – No DBT and DM for women with high MBD detected for the first time with DM. Tailored screening for MBD – Yes DBT for women with high MBD detected in previous screening exams. Guiding supplemental screening – No MRI, automated breast US system or HHUS for asymptomatic women with high MBD (unless already in practice).
	European Society of Breast Imaging (EUSOBI) [14]	2022	MBD notification – Yes “Should be informed on the diagnostic and prognostic implications of having dense breasts”. Guiding supplemental screening - Yes “SS with MRI at least every 4 years, preferably every 2–3 years for women with extremely dense breasts aged 50–70. If MRI screening is unavailable, US in combination with DM may be used”.
	The German Guideline Program in Oncology (German Cancer Society, German Cancer Aid, AWMF) [74] * Grade of recommendation: B - Recommendation using the language “should/should not”. Level of evidence: 3a -Systematic Review (with homogeneity*) of case-control studies. Consensus: Strong Consensus - >95 % of those entitled to vote ^#^ Grade of recommendation: B - Recommendation using the language “should/should not”. Level of evidence: 1b -Individual RCT (with narrow Confidence Interval). Consensus: Strong Consensus - >95 % of those entitled to vote	2021	Guiding supplemental screening - Yes SS with “US appears to be the most suitable method”. Improved sensitivity but lack of long-term evidence that it reduces mortality and “associated with a higher rate of biopsies than the national screening program”. ^#^ Use of tomosynthesis can increase sensitivity and “should be considered for testing in a quality assured programme”.
	The Gynaecological Oncology Working Group (AGO) [101] * Grade of recommendation: B - Consistent level 2 or 3 studies *or* extrapolations from level 1 studies. Level of evidence: 2a - Systematic review (with homogeneity) of cohort studies. AGO Grades of Recommendation: ++ - This investigation is highly beneficial for patients, can be recommended without restriction, and should be performed. ^#^ Grade of recommendation: B - Consistent level 2 or 3 studies *or* extrapolations from level 1 studies. Level of evidence: 1b - Individual randomised controlled trials (with narrow Confidence Interval). AGO Grades of Recommendation: ++ - This investigation is highly beneficial for patients, can be recommended without restriction, and should be performed.	2020	Guiding supplemental screening - Yes *Breast US for heterogeneously dense, extremely dense mammograms. ^#^MRI if screening mammogram is negative and breast composition extremely dense* 50–75 years old.
	The Royal College of Radiologists [33]	2019	Guiding supplemental screening – No Adjunctive US screening is not routinely recommended.
Outside the continent of Europe	The Royal Australian and New Zealand College of Radiologists [102]	2018	MBD reporting – Yes Formal report not issued in screening programmes in Australia or New Zealand.
	The Brazilian College of Radiology and Diagnostic Imaging (CBR), Brazilian Breast Disease Society (SBM), and Brazilian Federation of Gynecological and Obstetrical Associations (Febrasgo) [103] Recommendation based on reasonable scientific evidence, with a consistent consensus among the CBR, SBM, and Febrasgo that this recommendation should be strongly supported.	2017	Guiding supplemental screening - Yes Complementary US should be considered.
	Toward Optimized Practice (TOP) Working Group for Breast Cancer Screening [75]	2013	MBD specific screening interval “Annual DM may be suggested by radiologist if high MBD”. Inadequate outcome data for separate screening guideline for women with dense breasts. Guiding supplemental screening “US may have a role as determined by radiologist”.
	China Anti-Cancer Association [104] Level B recommendation: Based on studies with limited evidence or relatively consistent conclusions (Guideline development group moderately recommended).	2019	Guiding supplemental screening - Yes Breast US.
	The Japanese Breast Cancer Society [59], [60] *Strength of Recommendation (SoR): 3 Strength of Evidence (SoE): moderate, Consensus rate: 100 % ^#, $^SoR: 3, SoE: very weak, Consensus rate: 100 % ^$^SoE: Weak, Consensus rate: 100 %	2018	Guiding supplemental screening - No *Strongly recommended not to perform HHUS due to lack of evidence demonstrating mortality reduction, ^#^Weakly recommended not to perform DBT due to lack of evidence demonstrating mortality reduction, ^$^Strongly recommended not to use an automated whole-breast scanning system due to lack of evidence demonstrating mortality reduction.In voluntary screening, SS is a woman’s individual choice once she is provided with a sufficient explanation of the benefits and disadvantages regarding the supplemental screening modality. Guiding supplemental screening – No Lack of evidence demonstrating mortality reduction for contrast breast MRI and PET has not been established as a supplemental imaging modality for women with dense breasts. Contrast breast MRI and PET will be considered as future research questions. Information can be provided to women who attend voluntary breast cancer screening on the advantages and disadvantages of these modalities. MBD assessment – Yes MBD notification - No “Premature to uniformly notify. But if making the notification, municipalities are required to provide explanations and guidance.”
	Instance Nationale de l’Evaluation et de l’Accréditation en Santé (INEAS) and the Tunisian Society of Oncology [105] Conditional for either	2021	Guiding supplemental screening “Recommendation addressing SS with US modified from ‘conditional against’ to ‘conditional for either’ due to more favourable ratings by the adoloping panel in terms of equity and feasibility.”
	American College of Radiology [76] *Usually appropriate – “The imaging procedure is indicated in the specified clinical scenarios at a favorable risk-benefit ratio for patients” ^#, %, €^May be appropriate – “The imaging procedure may be indicated in the specified clinical scenarios as an alternative to imaging procedures with a more favorable risk-benefit ratio, or the risk-benefit ratio for patients is equivocal” ^$^May be appropriate (Disagreement)	2021	Guiding supplemental screening - Yes *DBT screening Guiding supplemental screening ^$^Insufficient evidence for or against US breast for average risk females with dense breasts but may be appropriate Guiding supplemental screening Limited or no relevant literature regarding the use of^#^Mammography with IV contrast ^%^MRI breast without and with IV contrast^€^MRI breast without and with IV contrast abbreviated as a supplemental screening modality for average risk females with dense breasts
	The American Academy of Family Physicians [77]	2021	Guiding supplemental screening Supports USPSTF recommendation
	American College of Obstetricians and Gynecologists [106]	2020	MBD notification “Compliance with state laws that may require disclosure to women of their MBD as recorded in a mammogram report.” Guiding supplemental screening - No For asymptomatic women with dense breasts and no other risk factors
	American Cancer Society [49], [78]	2007	Guiding supplemental screening **“**Insufficient evidence to recommend for or against breast MRI screening for women with heterogeneously or extremely dense breasts.”
	The National Comprehensive Cancer Network [77], [107]	2019	Guiding supplemental screening For women with dense breasts, there is insufficent evidence to recommend MRI. “US can increase cancer detection rate but can also result in an increase in recall rates and benign biopsies.” Molecular breast imaging is not recommended. Recommend counselling on the risks and benefits of SS
	The Society of Breast Imaging [79]	2010	Guiding supplemental screening US may be considered for women with dense breasts
	USPTSF [81], [108] Grade: I statement	2016	Guiding supplemental screening Insufficient evidence to assess the balance of benefits and harms of SS using breast US, MRI, DBT, or other methods

**Where information was not identified a dash (-) was used,** the following abbreviations were also used: MBD = Mammographic breast density, SS = Supplemental screening, IV= Intravenous DM = Digital Mammography, US = Ultrasound, HHUS = Hand-held Ultrasound, DBT = Digital Breast Tomosynthesis, MRI = Magnetic Resonance Imaging, PET = Positron emission tomography

### 3.5 Considerations for MBD policies in the screening setting

During the data extraction process, considerations relevant for MBD-related screening guidelines/recommendations were identified as shown in Table 4 and included those relating to the existing evidence base, resource requirements, availability of standardised MBD classification and measurement methods, supplemental screening modality, decision making, psychological impact, and access (Table 4).

**Table 4:** Summary of key factors and considerations relevant for the development and/or implementation of MBD related screening policies and guidance.

Key factors	Examples of considerations
Evidence base	Additional evidence is needed relating to: ● Impact of SS on long term breast cancer outcomes e.g. mortality, survival, and over diagnosis [59], [74] ● Impact of MBD notification without offering further specific care for those with dense breasts [30] ● Clinical and cost-effectiveness of MBD related guidelines within a PB screening programme [54]
Resources	Resources to be considered in the context of a screening programme include: ● Skilled expertise [62], [107] ● Screening capacity in terms of workforce, equipment, and software [49], [59], [109] ● Workforce planning [62], [79], [109] ● Cost to the healthcare system [20], [49], [59], [71], [73], [107] Impact of MBD related guidelines on the HCPs delivering the population-based screening programme in terms of: ● Their roles and responsibilities ● The demand on their time [73], [107] ● The need for additional education and training to provide such a service and to inform and support screening programme participants[30], [62], [63], [64]
MBD classification & measurement	Lack of a recommended standardised density measurement tool and criteria for use in a PB screening programme[53], [54], [74], [94] Considerations regarding: ● The optimal stage to start measuring breast cancer risk as MBD declines with age and menopause ● The optimal screening interval for screening programme participants with dense breasts and with non-dense breasts Factors that may influence MBD measures include: ● Differences between readers for the same screening phase or for different screening phases [44], [53], [54], [70] ● Differences between screening phases (as a woman’s age increases) [53], [54], [108]
Supplemental screening modality	Lack of consensus for optimum method Balance between increased sensitivity and decreased specificity of imaging modality with regard given to: ● False positives [44], [59], [63], [68], [70], [73], [74], [79], [106], [107], [108], [110] ● Recall rates [107] ● Biopsy rates [44], [71], [73], [74], [107], [110] ● Interval cancers [68] Operator dependency for some HHUS [62], [79], [107] Management of increased demand on PB screening programme due to resulting increased examination time and image interpretation time [49], [62], [107] Potential nephrotoxic side effects due to use of a contrast agent for MRI exams [59], [64], [98] Impact of non-breast incidental findings on healthcare system and the need for a management pathway for such findings [64], [98]
Decision making	The value of shared decision making between the HCPs and the screening programme client while taking into account: ● The individual screening programme client case [108] ● Contextualising relevant international evidence [93], [105], [108] The importance of accessible education for screening programme participants so that: ● They are informed of the benefits and limitations associated with each option available them in the clinical pathway ● They can appropriately contribute to the shared decision making process with HCPs
Psychological Impact	Potential psychological impact of MBD policies on screening programme participants: ● Increased anxiety [44], [53], [54], [71] ● Emotional distress [44] ● Confusion [53], [54]
Access	Heterogeneous provision of supplemental screening for screening programme participants with dense breasts outside the setting a population based screening programme can result in healthcare disparities amongst: ● Those who can afford these services and those who cannot [12], [30] ● Those who live in urban areas and those who live in rural areas [12]

The following abbreviations were also used: HCPs= Healthcare professionals, SS = Supplemental screening, MBD = Mammographic breast density, PB = Population-based, US = Ultrasound, MRI = Magnetic Resonance Imaging

## 4 Discussion

This scoping review provides an overview of literature relating to MBD practices in BSPs and recommendations from professional societies and GDGs internationally and summarises current guidance relating to MBD assessment, reporting, notification, or use for guiding supplemental screening. The findings described differing approaches regarding practices and guidelines incorporating MBD from BSPs, professional societies, and GDGs; highlighting the absence of consensus currently surrounding optimal practices for women with dense breasts in breast cancer screening.

Regarding MBD assessment, it was found that for screening programme participants who attend population-based BSPs in Austria, the Czech Republic, and a regional BSP in Denmark, MBD is assessed as part of routine breast screening [29], [34], [35], [47], [48], [49], [50]. Outside of Europe, MBD assessment practices were identified for regions in Australia, Canada, and in Taiwan [12], [37], [38], [39], [40], [41], [42], [43], [44], [45], [51]. Typically, MBD can be assessed visually, using semi-automated tools, or using fully automated tools [52]. However, in this review we were unable to identify any recommendations or practices which mentioned a specific semi-automated or automated MBD measurement tool currently being used. However, the ACR BI-RADS Breast Composition Categories were most frequently mentioned, with the population-based BSP in Austria and the regional BSPs in Denmark, Australia, and Canada using these categories [35], [37], [41], [47]. Similarly, the ECIBC include the BI-RADS categories (III and IV) along with quantitative percent MBD values (50–75 % and >75 %) and Wolfe categories (P2 and DY) to define dense breasts for their guidance [15], [19], [20], [21], [22]. Challenges still remain as identified, regarding the reproducibility of MBD measures between readers and between screening phases [53], [54]. As noted by the UK National Screening Committee, within the context of a population-based BSP, a reliable and reproducible MBD measurement tool is needed. Advancements in the area with the potential to overcome these challenges include the incorporation of artificial intelligence algorithms[55], [56].

In relation to the reporting of MBD, less information was identified. In the context of population-based BSPs within Europe, the findings of this present review only found Austria and the Czech Republic included the reporting of MBD as part of routine breast screening [29], [34], [47], [48], [49], [50]. Literature specifically mentioning MBD reporting practices were also noted in Taiwan and in regions of Australia and Canada [12], [37], [38], [39], [40], [41], [42], [43], [44], [45], [51].

While no literature was found describing MBD notification practices in BSPs within Europe, the EUSOBI recently recommended informing participants attending breast screening about their MBD [14]. Outside Europe, some regional BSPs in Australia and Canada notify women with dense breasts [38], [41], [44], [45]. In Western Australia, MBD notification policy is incorporated within BreastScreen Western Australia and this information is included supplementary to their mammography report and a copy is shared with the woman’s general practitioner, though supplemental screening is not offered with this notification to women with dense breasts (MBD > 50%) [38]. While in Canada, MBD notification policies are used to inform the screening interval for BSP participants; women with MBD ≥ 75% are offered more frequent screening (every 12–18 months) [41], [44], [45]. However, in the USA, state specific differences were largely determined by legislation, with legislation in place in 38 states for MBD notification [10], [57]. Amendments to the Food and Drug Administration Mammography Quality Standards Act specific to MBD reporting and notification were recently published in 2023 [11]. These updates which will be effective as of September 10th 2024 will require MBD assessment information using the 5th edition BI-RADS categories to be included in mammography reports shared with healthcare providers and that women are informed whether their breast tissue is dense or not dense [58]. In addition, the FDA provide specific language to be used in the lay summary of a woman’s mammography results which highlight the masking effect of dense tissue for mammography and to encourage shared and informed decision making [58]. While the Japanese Breast Cancer Society do not recommend MBD notification, they do acknowledge the need of providing appropriate information and guidance if women with dense breasts are notified [59], [60]. Regarding the communication of MBD information, literature in the area demonstrates the variation in information communicated and that often this information is poorly understood [61]. Lack of understanding can contribute to feelings of increased anxiety, confusion, and emotional distress experienced by BSP participants [44], [53], [54]. While accessible information is imperative for BSP participants so that they are well informed to contribute to the shared decision making process, there is also a need for additional education and training for healthcare professionals within a population-based BSP to inform and support BSP participants [30], [62], [63], [64].

Literature was identified from population-based and regional-based BSPs in Austria, Czech Republic, France, Germany, Greece, Spain, and Sweden that describe supplemental screening practices with ultrasound and DBT for women with dense breasts or shorter screening intervals for younger women [29], [34], [36], [46], [47], [48], [49], [50], [62], [63], [65], [66], [67]. Within Europe, current recommendations from the ECIBC GDG recommend the use of DBT for tailored screening for asymptomatic women with high MBD previously detected [15]. In Germany, it was noted by the Institute for Quality and Efficiency in Health Care, the national health technology assessment body, that typically women are not notified about their MBD but that women can be advised by their doctor to undergo supplemental screening out of pocket with ultrasound or MRI [63], [67]. In the UK, MBD is currently not incorporated in the breast screening pathway as part of their national BSP and in a systematic review commissioned by the UK National Screening Committee, it was determined that there was insufficient evidence to balance the risks, benefits, and costs associated with ultrasound after negative mammography for women with dense breasts [53]. Outside of Europe, in some regional Canadian BSPs, women with dense breasts may be offered supplemental ultrasound examinations [12], [41], [42], [43]. In comparison in the USA, while there is no organised BSP, the provision of supplemental screening for women with dense breasts is currently mandated in five states with legislation in place regarding insurance coverage [10]. Factors such as the evidence base for informing clinical practices, resource requirements, optimum supplemental screening modalities, and associated considerations such as workforce planning, equipment, and the balance between the sensitivity and specificity of the imaging modality were acknowledged in guidance from the Health Council of the Netherlands, the EUSOBI, and the ECIBC GDG [14], [20], [68]. Recent recommendations by EUSOBI and the Health Council of the Netherlands both incorporated results from the DENSE trial in their guidance, with the EUSOBI recommending informing BSP participants about their MBD and the provision of supplemental screening with MRI for women with extremely dense breasts (ACR BI-RADS category D) aged 50–70 years every 2–4 years [14], [25], [68]. In contrast the Health Council of the Netherlands currently state that “supplemental MRI screening should not be included” in the BSP, for women with extremely dense breast tissue [68]. Both organisations acknowledge that a significant investment in resources would be needed to implement supplemental screening MRI for women with extremely dense breasts in the population based breast screening setting. Notably, the Health Council of the Netherlands consider it an inefficient investment as the “benefits barely outweigh the drawbacks” [68]. While a study conducted by Geuzinge et al. using results from the DENSE trial found that screening with MRI every four years for women with extremely dense breasts was cost effective, they also acknowledged that such a screening strategy would require investment in MRI machines, medical physicists, radiographers, and radiologists. In addition, if such a screening strategy was implemented it could potentially present capacity challenges along with inequitable access if women choose to avail of opportunistic screening [69]. These variations in recommendations from different organisations highlight the lack of consensus surrounding the optimum role of MBD in the population-based screening setting and supplemental screening modality for women with dense breasts. It is also important to note that the guidance from both organisations focussed on women with extremely dense breasts (ACR BI-RADS D) and we were unable to identify any guidance for women whose MBD is categorised ACR BI-RADS C. Further research on the benefits of supplemental screening among women in this category is warranted.

The importance of a strong evidence base is imperative for informing decision making processes. However, in two BSPs and four recommendations from professional organisations published from 2016 onwards, it was concluded that there was insufficient or limited evidence to make a recommendation [43], [49], [70], [71], [72], [73], [74], [75], [76], [77], [78], [79], [80], [81]. These findings highlight the need for further research in the area. Recent recommendations from the Japanese Breast Cancer Society and the National Comprehensive Cancer Network refer to a more individualised approach, as opposed to a single population-based approach where women make a shared decision with their healthcare professional upon being informed of the risks and benefits of their options [59], [82]. Personalised breast screening aims to overcome the limitations of the current age-based approach adopted by BSPs and determine an appropriate tailored screening approach based on a woman’s individual risk [83], [84]. Current ongoing clinical trials include the international randomised controlled study, My Personal Breast Screening (MyPeBS), which is assessing the effectiveness of an individual risk based approach compared to the standard approach in the different study sites [85], [86]. MBD is considered in both arms of the study and is also incorporated into the risk calculations conducted as part of the intervention [85], [86]. In the USA, the Women Informed to Screen Depending On Measures of risk (WISDOM) study is comparing annual screening to a personalised screening approach incorporating MBD, lifestyle risk factors, and genetic risk factors in the risk assessments [87]. Further, the UK based Breast Screening – Risk Adaptive Imaging for Density (BRAID) trial aims to evaluate different supplemental screening modalities for women with dense breasts participating in a BSP [88]. Findings from these studies will play a significant role in informing personalised screening approaches in the context of BSPs; adding to the evidence base to establish an optimal supplemental screening modality for women with dense breasts within the population based screening setting. Results are also expected from the ongoing study within BreastScreen South Australia, which is examining the measurement, reporting, and notification of MBD to BSP participants [89]. In addition, an update of the 2016 United States Preventive Services Taskforce Breast Cancer: Screening recommendation is currently in progress [90]. MBD will also be considered in the next update of the breast cancer screening recommendations for women at increased risk by the American Cancer Society [91]. At the time of writing, recommendations to increase the target age range to 45–74 years for breast screening and the consideration of MRI where medically appropriate are included in the 2022 European Council recommendation 2022/C 473/01 replacing the 2003 European Council recommendation 2003/878/EC [92]. Evidence-based breast screening policies, recommendations, and clinical guidelines will continue to evolve over time as more quality research is conducted and evidence becomes available.

The findings presented were based on the literature identified through the search approaches undertaken and thus while efforts were made to ensure a comprehensive search, it is feasible that not all relevant documents may have been captured. Limitations associated with this scoping review include the possibility that relevant materials may not have been identified or were published after the electronic database and grey literature searches were conducted. Some documents were published prior to 2010 and may no longer reflect the current position of an organisation [78]. When selecting countries for inclusion in the targeted search, we opted to include geographical areas which were known to offer breast screening services. As this is a scoping review, a critical appraisal of the documents selected for inclusion was not conducted as this is not part of the process [26].

## 5 Conclusion

This scoping review identified literature relating to practices and guidelines incorporating MBD for assessment, reporting, notification, and guiding supplemental screening from international BSPs, professional societies, and GDGs. It highlights the current absence of consensus regarding the clinical role of MBD in international BSPs. Further research needed in this area includes building an evidence base on the impact of MBD-related practices on short and long term breast cancer outcomes such as breast cancer detection, mortality, and survival to contribute to the current evidence base for future policy and recommendations. Additional research is also needed to further understand how international guidance can be implemented in national breast screening settings with consideration given to the further training and education needed by healthcare professionals. Advances in technology with artificial intelligence, along with the implementation of a reproducible, standardised method to measure and report MBD, and the establishment of an optimal supplemental screening modality that is both clinical and cost-effective will be key to incorporating MBD practices in population-based BSPs.

## Ethics approval

As this is a scoping review of existing literature, ethical approval was not required.

## Funding

This work was supported by the 10.13039/100010414Health Research Board (HRB) of Ireland through the HRB Emerging Investigator Award [EIA-2019–012]. The opinions, findings and conclusions or recommendations expressed in this material are those of the authors and do not necessarily reflect the view of the HRB.

## CRediT authorship contribution statement

**Jessica O’Driscoll:** Conceptualization, Investigation, Methodology, Project Administration, Visualisation, Writing – Original Draft Preparation, Writing – Review & Editing. **Aileen Burke:** Investigation, Writing – review & editing. **Therese Mooney:** Writing – review & editing. **Niall Phelan:** Writing – review & editing. **Paola Baldelli:** Writing – review & editing. **Alan Smith:** Writing – review & editing. **Patricia Fitzpatrick:** Writing – review & editing. **Kathleen Bennett:** Conceptualization, Methodology, Supervision, Writing – review & editing. **Fidelma Flanagan:** Writing – review & editing. **Maeve Mullooly:** Conceptualization, Funding acquisition, Methodology, Project administration, Supervision, Writing – original draft, Writing – review & editing.

## Declaration of Competing Interest

The authors declare that they have no known competing financial interests or personal relationships that could have appeared to influence the work reported in this paper.
