# Supplementary material for: A scoping review of programme specific mammographic breast density related guidelines and practices within breast screening programmes
Source: Eur J Radiol Open. 2023 Aug 2;11:100510. doi: 10.1016/j.ejro.2023.100510 (PMC10407884; doi:10.1016/j.ejro.2023.100510)
Supplement: Supplementary file 1 — Supplementary material [file mmc1.docx]

**Article title:** A scoping review of programme specific mammographic breast density related guidelines and practices within breast screening programmes

**Author names:** Jessica O’Driscoll, Aileen Burke, Therese Mooney, Niall Phelan, Paola Baldelli, Alan Smith, Suzanne Lynch, Patricia Fitzpatrick, Kathleen Bennett, Fidelma Flanagan, Maeve Mullooly

**Corresponding author:** Miss Jessica O’Driscoll, School of Population Health, RCSI University of Medicine and Health Sciences, Beaux Lane House, Mercer St. Lower, Dublin 2, Ireland. [JessicaODriscol20@rcsi.com](mailto:JessicaODriscol20@rcsi.com)

**Online Resource** **1 - Exploring the use of programme specific mammographic breast density related guidelines and practices within population breast screening programmes: A scoping review protocol**

***Objective***

The primary objective of this scoping review is to identify and describe current programme specific MBD related guidelines and practices in breast screening programmes.

**Methods**

***Study design***

A scoping review will be used to address the research question and will employ the framework proposed by the Joanna Briggs Institute (JBI) guidelines for scoping reviews which builds upon the original framework by Arksey and O’Malley [1, 2]. This review will be reported according to the Preferred Reporting Items for Systematic Reviews and Meta-analyses extension for Scoping Reviews (PRISMA-ScR) guidelines [3].

***Stage 1: Identifying the research question***

Using the Population, Concept and Context (PCC) mnemonic recommended in the JBI guidelines for scoping reviews [1], the following primary research question was identified: What are the current programme specific MBD related guidelines and practices (*Concept*) for women attending routine breast screening (*Population*), within organised breast screening programmes (*Context*)?

In addition, sub-questions exploring the Concept and Context elements to assist with evidence mapping are outlined below:

1. What are the current programme specific MBD related guidelines and practices (relating to MBD assessment, reporting, notifying, and use for guiding supplemental screening) for women attending routine breast screening, within European breast screening programmes?
2. What are the current programme specific MBD related guidelines and practices (relating to MBD assessment, reporting, notifying, and use for guiding supplemental screening) for women attending routine breast screening, within breast screening programmes outside Europe?
3. What considerations are important in the development and implementation of MBD related screening guidelines and practices for women attending routine breast screening within breast screening programmes?

***Stage 2: Identifying relevant studies***

In order to identify relevant literature, a comprehensive three-step search strategy as proposed by the JBI guidelines, will be conducted [1].

*Step 1:* An initial search of the electronic databases, MEDLINE (PubMed) and EMBASE will be carried out. Descriptive terms used in the titles and abstracts of retrieved articles and the subject headings used to describe such articles will be analysed.

*Step 2:* Using the identified descriptive terms and subject headings and upon consultation with a medical librarian (NOB), a comprehensive final search strategy will be developed. The search strategy will be adapted for searches in each of the following electronic databases: MEDLINE (PubMed), EMBASE, CINAHL Plus, Scopus and Web of Science. The search strategy for MEDLINE (PubMed) is set out in Table 1. As screening guidelines and practices may not be reported in peer-reviewed articles, the grey literature search plan employed by Godin et al., will be adapted for this review [4]. The search strategy will be modified to search grey literature databases such as OpenGrey.eu, NICE evidence, and Lenus, the Irish Health Repository. Search engines such as Bielefeld Academic Search Engine, Mednar, and the custom Google search engine for official government resources provided by the University of Toronto Libraries will also be searched. A targeted search of websites such as those of international breast screening programmes and government organisations will also be conducted.

*Step 3:* The reference lists of all retrieved articles and documents will also be searched for any additional relevant articles.

***Stage 3: Study selection***

All articles and documents identified through the searches will be imported into Endnote X9 and duplicates removed. Remaining references will then be imported into the online systematic review management tool, Rayyan (<https://www.rayyan.ai>). Titles and abstracts will be screened independently for inclusion by two reviewers using the pre-specified inclusion and exclusion criteria listed in Table 2. For documents where an abstract is not available, executive summaries and the table of contents will be screened. Relevant articles and documents will then undergo full-text review to determine their inclusion in the review by one reviewer and a second reviewer will independently review a random 20% of the articles and documents. Reasons for the exclusion of articles and documents will also be noted. Any disagreements will be resolved by consensus and if necessary, a third reviewer will be consulted. The study selection process will be presented in a PRISMA flow diagram along with a narrative description of the process [3].

***Stage 4: Charting the data***

The template data extraction form provided by the JBI will be adapted for this review [1]. The data extraction form will be piloted on three retrieved articles to ensure all relevant data is charted and will be adapted if necessary. Data will be extracted by one reviewer. The following information will be captured:

1. Study details: Title, author and year of publication.
2. Screening programme characteristics: Type of breast screening programme setting (population-based, regional, or organised), location, date of programme commencement, screening interval, screening method, use of double reading, affordability (free, covered by public health insurance, or co-payment), individual invitation, age standardised incidence rate, attendance rate, screening coverage, and recall rate.
3. Population: Target age range and risk profile (average or intermediate risk).
4. Concept: Guidance type (screening programme, government agency, or professional society), guidance development methods/source, screening guidance for: dense breasts, MBD assessment, MBD reporting, MBD notification, supplemental screening, and development/implementation considerations for such guidance.

***Stage 5: Collating, summarising and reporting the results***

Results from the scoping review will be reported according to the PRISMA-ScR guidelines [3]. Data mapping will be guided by the research questions. A narrative summary aligning the summarised results with the scoping review objective and research questions will be performed. A potential limitation of this study relates to literature published in non-English languages. However, efforts will be made to overcome any language barriers using an approach adapted from Rockliffe’s recent discussion paper [5]. Google Translate will be used to translate non-English language articles and documents. Potential knowledge gaps and suggestions for future research will be outlined, as well as the strengths and limitations of this scoping review. Any deviations from this scoping review protocol will also be recorded with the associated reason for the deviation.

**References**

1. Chapter 11: Scoping reviews - JBI Manual for Evidence Synthesis - JBI GLOBAL WIKI [<https://wiki.jbi.global/display/MANUAL/Chapter+11%3A+Scoping+reviews>]

2. Arksey H, O'Malley L: Scoping studies: towards a methodological framework. *International journal of social research methodology* 2005, 8(1):19-32.

3. Tricco AC, Lillie E, Zarin W, O'Brien KK, Colquhoun H, Levac D, Moher D, Peters MDJ, Horsley T, Weeks L *et al*: PRISMA Extension for Scoping Reviews (PRISMA-ScR): Checklist and Explanation. *Annals of Internal Medicine* 2018, 169(7):467-473.

4. Godin K, Stapleton J, Kirkpatrick SI, Hanning RM, Leatherdale ST: Applying systematic review search methods to the grey literature: a case study examining guidelines for school-based breakfast programs in Canada. *Systematic Reviews* 2015, 4(1):138.

5. Rockliffe L: Including non-English language articles in systematic reviews: A reflection on processes for identifying low-cost sources of translation support. *Research Synthesis Methods* 2022, 13(1):2-5.

**Tables**

Table 1: The search strategy for the electronic database MEDLINE (PubMed).

| **Number** | **Search terms** |
| --- | --- |
| 1 | Breast neoplasms [MeSH] |
| 2 | (Breast OR Mamma*) AND (Cancer* OR Neoplasm* OR Carcino* OR Tumo* OR Malignanc*) |
| 3 | #1 OR #2 |
| 4 | Mass screening [MeSH] |
| 5 | Early detection of cancer [MeSH] |
| 6 | Mammography [MeSH] |
| 7 | Screen* OR (Early AND (detect* OR diagnos*)) |
| 8 | #4 OR #5 OR #6 OR #7 |
| 9 | #3 AND #8 |
| 10 | Breast density [MeSH] |
| 11 | ((mammogr* OR breast OR tissue OR mammary) AND (densit* OR parenchym*)) OR Mammogr* breast densit* OR Dense breast* |
| 12 | #10 OR #11 |
| 13 | Classification [MeSH] |
| 14 | Assessment OR Classification |
| 15 | #13 OR #14 |
| 16 | Legislation & jurisprudence [MeSH] |
| 17 | Report* OR Notification |
| 18 | #16 OR #17 |
| 19 | Magnetic Resonance Imaging [MeSH] |
| 20 | Mammography [MeSH] |
| 21 | Imaging, Three-dimensional [MeSH] |
| 22 | Ultrasonography, mammary [MeSH] |
| 23 | ((Supplement* OR Adjunc* OR Addition* OR Plus) And Screen*) OR Ultras* OR Tomo* OR MRI OR Magnetic resonance imag* |
| 24 | #19 OR #20 OR #21 OR #22 OR #23 |
| 25 | #15 OR #18 OR #24 |
| 26 | #12 AND #25 |
| 27 | Practice guideline [MeSH] |
| 28 | Practice guideline as topic [MeSH] |
| 29 | Guideline adherence [MeSH] |
| 30 | Guidelines as topic [MeSH] |
| 31 | Consensus [MeSH] |
| 32 | Evidence-based medicine [MeSH] |
| 33 | Practice guideline* OR guideline* OR Recommendation* OR Position statement* OR Consensus OR Polic* OR Legislation* |
| 34 | #27 OR #28 OR #29 OR #30 OR #31 OR #32 OR #33 |
| 35 | #9 AND #34 |
| 36 | #35 AND #26 |

Table 2: Inclusion and exclusion criteria for the review.

| **Criterion** | **Inclusion Criteria** | **Exclusion Criteria** |
| --- | --- | --- |
| Population | Include documents that identify guidelines, recommendations, or position statements relating to women eligible to attend a breast cancer screening programme i.e. asymptomatic, within the target age range, and have an average risk of breast cancer. | Exclude documents that identify guidelines, recommendations, or position statements relating to women who would not be eligible to attend a breast cancer screening programme i.e. women who are symptomatic, outside the target age range, or have a high risk of breast cancer. |
| Concept | Include documents that identify guidelines, recommendations, or position statements for the purpose of MBD assessment, reporting, notifying, or use for guiding supplemental screening.  Include documents that identify considerations important in the development and implementation of MBD related screening guidelines. | Exclude documents that identify guidelines, recommendations, or position statements for any other purpose. |
| Context | Include documents that identify guidelines, recommendations, or position statements for organised screening settings. | Exclude documents that identify guidelines, recommendations, or position statements for opportunistic screening settings. |
| Location | No geographical restrictions | - |
| Time period | No time-period restrictions | - |
| Language | No language restrictions | - |
