# Supplementary material for: A scoping review of programme specific mammographic breast density related guidelines and practices within breast screening programmes
Source: Eur J Radiol Open. 2023 Aug 2;11:100510. doi: 10.1016/j.ejro.2023.100510 (PMC10407884; doi:10.1016/j.ejro.2023.100510)
Supplement: Supplementary file 3 — Supplementary material [file mmc3.docx]

**Article title:** A scoping review of programme specific mammographic breast density related guidelines and practices within breast screening programmes

**Author names:** Jessica O’Driscoll, Aileen Burke, Therese Mooney, Niall Phelan, Paola Baldelli, Alan Smith, Suzanne Lynch, Patricia Fitzpatrick, Kathleen Bennett, Fidelma Flanagan, Maeve Mullooly

**Corresponding author:** Miss Jessica O’Driscoll, School of Population Health, RCSI University of Medicine and Health Sciences, Beaux Lane House, Mercer St. Lower, Dublin 2, Ireland. [JessicaODriscol20@rcsi.com](mailto:JessicaODriscol20@rcsi.com)

**Online Resource** **3 - Electronic database search strategies**

**MEDLINE (PubMed)**

| **Number** | **Search terms** |
| --- | --- |
| 1 | Breast neoplasms [MeSH] |
| 2 | (Breast OR Mamma*) AND (Cancer* OR Neoplasm* OR Carcino* OR Tumo* OR Malignanc*) |
| 3 | #1 OR #2 Query #5 |
| 4 | Mass screening [MeSH] |
| 5 | Early detection of cancer [MeSH] |
| 6 | Mammography [MeSH] |
| 7 | Screen* OR (Early AND (detect* OR diagnos*)) |
| 8 | #4 OR #5 OR #6 OR #7 Query #12 |
| 9 | #3 AND #8 Query #13 |
| 10 | Breast density [MeSH] |
| 11 | ((mammogr* OR breast OR tissue OR mammary) AND (densit* OR parenchym*)) OR Mammogr* breast densit* OR Dense breast* |
| 12 | #10 OR #11 Query #20 |
| 13 | Classification [MeSH] |
| 14 | Assessment OR Classification |
| 15 | #13 OR #14 Query #23 |
| 16 | Legislation & jurisprudence [MeSH] |
| 17 | Report* OR Notification |
| 18 | #16 OR #17 Query #26 |
| 19 | Magnetic Resonance Imaging [MeSH] |
| 20 | Mammography [MeSH] |
| 21 | Imaging, Three-dimensional [MeSH] |
| 22 | Ultrasonography, mammary [MeSH] |
| 23 | ((Supplement* OR Adjunc* OR Addition* OR Plus) And Screen*) OR Ultras* OR Tomo* OR MRI OR Magnetic resonance imag* |
| 24 | #19 OR #20 OR #21 OR #22 OR #23 Query #33 |
| 25 | #15 OR #18 OR #24 Query #34 |
| 26 | #12 AND #25 Query #35 |
| 27 | Practice guideline [MeSH] |
| 28 | Practice guideline as topic [MeSH] |
| 29 | Guideline adherence [MeSH] |
| 30 | Guidelines as topic [MeSH] |
| 31 | Consensus [MeSH] |
| 32 | Evidence-based medicine [MeSH] |
| 33 | Practice guideline* OR guideline* OR Recommendation* OR Position statement* OR Consensus OR Polic* OR Legislation* |
| 34 | #27 OR #28 OR #29 OR #30 OR #31 OR #32 OR #33 Query #38 |
| 35 | #9 AND #34 Query #39 |
| 36 | #35 AND #26 Query #40 |

**CINAHL Plus**

| **Number** | **Search terms** |
| --- | --- |
| 1 | Breast tumor [Subject heading] |
| 2 | (Breast OR Mamma*) AND (Cancer* OR Neoplasm* OR Carcino* OR Tumo* OR Malignanc*) |
| 3 | #1 OR #2 Query #5 |
| 4 | Mass screening [Subject heading] |
| 5 | Early cancer diagnosis [Subject heading] |
| 6 | Mammography [Subject heading] |
| 7 | Screen* OR (Early AND (detect* OR diagnos*)) |
| 8 | #4 OR #5 OR #6 OR #7 Query #8 |
| 9 | #3 AND #8 Query #9 |
| 10 | Breast density [Subject heading] |
| 11 | ((mammogr* OR breast OR tissue OR mammary) AND (densit* OR parenchym*)) OR Mammogr* breast densit* OR Dense breast* |
| 12 | #10 OR #11 Query #16 |
| 13 | Classification [Subject heading] |
| 14 | Assessment OR Classification |
| 15 | #13 OR #14 Query #19 |
| 16 | Legislation [Subject heading] |
| 17 | Report* OR Notification |
| 18 | #16 OR #17 Query #22 |
| 19 | Magnetic Resonance Imaging [Subject heading] |
| 20 | Mammography [Subject heading] |
| 21 | Imaging, Three-dimensional [Subject heading] |
| 22 | Ultrasonography, mammary [Subject heading] |
| 23 | ((Supplement* OR Adjunc* OR Addition* OR Plus) And Screen*) OR Ultras* OR Tomo* OR MRI OR Magnetic resonance imag* |
| 24 | #19 OR #20 OR #21 OR #22 OR #23 Query #29 |
| 25 | #15 OR #18 OR #24 Query #30 |
| 26 | #12 AND #25 Query #31 |
| 27 | Practice guideline [Subject heading] |
| 28 | Guideline adherence [Subject heading] |
| 29 | Consensus [Subject heading] |
| 30 | Evidence-based medicine [Subject heading] |
| 31 | Practice guideline* OR guideline* OR Recommendation* OR Position statement* OR Consensus OR Polic* OR Legislation* |
| 32 | #27 OR #28 OR #29 OR #30 OR #31 OR #32 OR #33 Query #34 |
| 33 | #9 AND #34 Query #35 |
| 34 | #35 AND #26 Query #36 |

**Scopus**

| **Number** | **Search terms** |
| --- | --- |
| 1 | (Breast OR Mamma*) AND (Cancer* OR Neoplasm* OR Carcino* OR Tumo* OR Malignanc*) |
| 2 | Screen* OR (Early AND (detect* OR diagnos*)) |
| 3 | #1 AND #2 |
| 4 | ((mammogr* OR breast OR tissue OR mammary) AND (densit* OR parenchym*)) OR Mammogr* breast densit* OR Dense breast* |
| 5 | Assessment OR Classification |
| 6 | Report* OR Notification |
| 7 | ((Supplement* OR Adjunc* OR Addition* OR Plus) And Screen*) OR Ultras* OR Tomo* OR MRI OR Magnetic resonance imag* |
| 8 | #5 OR #6 OR #7 |
| 9 | #4 AND #8 |
| 10 | Practice guideline* OR guideline* OR Recommendation* OR Position statement* OR Consensus OR Polic* OR Legislation* |
| 11 | #3 AND #10 |
| 12 | #11 AND #26 Query #40 |

**EMBASE**

| **Number** | **Search terms** |
| --- | --- |
| 1 | Breast tumor [Emtree] |
| 2 | (Breast OR Mamma*) AND (Cancer* OR Neoplasm* OR Carcino* OR Tumo* OR Malignanc*) |
| 3 | #1 OR #2 |
| 4 | Mass screening [Emtree] OR Early cancer diagnosis [Emtree] OR Mammography [Emtree] |
| 5 | Screen* OR (Early AND (detect* OR diagnos*)) |
| 6 | #4 OR #5 |
| 7 | #3 AND #6 |
| 8 | Breast density [Emtree] |
| 9 | ((mammogr* OR breast OR tissue OR mammary) AND (densit* OR parenchym*)) OR Mammogr* breast densit* OR Dense breast* |
| 10 | #8 OR #9 |
| 11 | Classification [Emtree] OR Breast imaging reporting and data system [Emtree] |
| 12 | Assessment OR Classification |
| 13 | #11 OR #12 |
| 14 | Legislation & jurisprudence [Emtree] |
| 15 | Report* OR Notification |
| 16 | #14 OR #15 |
| 17 | Nuclear Magnetic Resonance Imaging [Emtree] OR Digital breast tomosynthesis [Emtree] OR Echomammography [Emtree] OR Mammography [Emtree] |
| 18 | ((Supplement* OR Adjunc* OR Addition* OR Plus) And Screen*) OR Ultras* OR Tomo* OR MRI OR Magnetic resonance imag* |
| 19 | #17 OR #18 |
| 20 | #13 OR #16 OR #19 |
| 21 | #10 AND #20 |
| 22 | Practice guideline [Emtree] OR Consensus [Emtree] OR Evidence-based medicine [Emtree] OR Health care policy [Emtree] |
| 23 | Practice guideline* OR guideline* OR Recommendation* OR Position statement* OR Consensus OR Polic* OR Legislation* |
| 24 | #22 OR #23 |
| 25 | #7 AND #24 |
| 26 | #25 AND #21 |

**Web of Science**

| **Number** | **Search terms** |
| --- | --- |
| 1 | (Breast OR Mamma*) AND (Cancer* OR Neoplasm* OR Carcino* OR Tumo* OR Malignanc*) |
| 2 | Screen* OR (Early AND (detect* OR diagnos*)) |
| 3 | #1 AND #2 |
| 4 | ((mammogr* OR breast OR tissue OR mammary) AND (densit* OR parenchym*)) OR Mammogr* breast densit* OR Dense breast* |
| 5 | Assessment OR Classification |
| 6 | Report* OR Notification |
| 7 | ((Supplement* OR Adjunc* OR Addition* OR Plus) And Screen*) OR Ultras* OR Tomo* OR MRI OR Magnetic resonance imag* |
| 8 | #5 OR #6 OR #7 |
| 9 | #4 AND #8 |
| 10 | Practice guideline* OR guideline* OR Recommendation* OR Position statement* OR Consensus OR Polic* OR Legislation* |
| 11 | #3 AND #10 |
| 12 | #11 AND #9 |
